# Correlates of Maternal Depressive Symptomatology Among Latina Head Start Mothers

**DOI:** 10.1007/s10995-026-04253-4

**Published:** 2026-04-10

**Authors:** Abigail Palmer Molina, Elizabeth M. Premo, Lei Duan, Ferol E. Mennen

**Affiliations:** 1 University of Chicago Crown Family School of Social Work, Policy, and Practice, Chicago, IL USA; 2 University of Wisconsin-Madison Sandra Rosenbaum School of Social Work, Madison, WI USA; 3 Suzanne Dworak-Peck School of Social Work, University of Southern California, Los Angeles, CA USA

**Keywords:** Latina, Maternal depression, Early childhood, Head Start, Correlates

## Abstract

**Objectives:**

Low-income, Latina mothers are at high risk for experiencing maternal depression, which can impact their young children. This study sought to identify socioeconomic, psychosocial, health, and cultural correlates of depressive symptoms among Latina mothers of Head Start children.

**Methods:**

A cross-sectional study was conducted with baseline data from 163 Latina mothers enrolled in a larger intervention trial (*M*_age_ = 32.7, *SD*=6.3). Mothers were screened for depressive symptoms using the Center for Epidemiology Studies Depression Scale (CES-D). Hierarchical regression models examined whether socioeconomic factors (education, employment, insurance status, income, economic pressure, and number of children in household), psychosocial and health factors (age, lifetime trauma exposure, intimate partner violence (IPV) exposure, living situation, health problems, social support, and prior mental health history), and cultural factors (maternal nativity, preferred language) explained a statistically significant amount of variance in maternal depressive symptoms.

**Results:**

38% of Latina Head Start mothers reported clinically significant levels of depressive symptomatology. Reporting income from earnings and social support were negatively associated with depressive symptoms, whereas lifetime trauma exposure, exposure to current physical and verbal IPV, and endorsing a history of depression were positively associated with depressive symptoms. Cultural factors were not significant.

**Conclusions for Practice:**

Findings indicate a high rate of elevated depressive symptomatology in this sample. Cross-system interventions are needed that address both structural and psychosocial contributors to maternal depression for low-income, Latina mothers of young children. Future research should continue to explore the role of cultural factors for Latina mothers.

**Supplementary Information:**

The online version contains supplementary material available at 10.1007/s10995-026-04253-4.

## Significance

Latina Head Start mothers are at high risk for maternal depression. This study found that both structural (e.g., income) and psychosocial factors (e.g., social support, trauma exposure, and prior mental health history) were associated with maternal depressive symptoms for this population. Multidimensional interventions that address both structural and psychosocial factors are needed.

**Supplementary Information:**

The online version contains supplementary material available at 10.1007/s10995-026-04253-4.

## Introduction

Maternal depression is associated with negative outcomes for children during the early childhood period. Experiencing depression can impede maternal responsiveness (Lovejoy et al., 2000), and children of depressed mothers are at increased risk for deficits in their social-emotional (Goodman et al., 2011; Sutherland et al., 2021), cognitive (Liu et al., 2017), and physical development (Thompson et al., 2018). Latina mothers in the United States are at high risk for experiencing depression, with studies reporting rates ranging from 12 to 59% in the postpartum period^1^ (Blackmore & Chaudron 2014; Lucero et al., 2012; Luis Sanchez et al., 2020). A growing body of research has focused on correlates of maternal depressive symptoms among Latina mothers during postpartum, recognizing that Latina mothers often experience compounding stressors related to their multiple marginalized identities. However, there is a lack of literature focused on Latina mothers of preschool age children. This population warrants further study since there is a high prevalence of elevated maternal depressive symptoms during the preschool period for low-income mothers (Baker, 2018; Lanzi et al., 1999; Woolhouse et al., 2015). Low-income Latina mothers may also continue to experience many of the same economic and psychosocial stressors past the postpartum period.

Lara-Cinisomo et al. (2016) proposed a biopsychosocial conceptual framework for postpartum depression risk among Latina mothers that elucidates the importance of contextual and culturally relevant contributors for this population, including poverty, traumatic experiences, racial discrimination, and acculturation and acculturative stress (2016). One prior empirical test of the contextual and cultural stressors outlined by this model found that domestic violence and discrimination were associated with higher depressive symptoms postpartum for low-income Latina mothers, but foreign-born status and poverty were not (Ponting et al., 2020). Further research is needed to elucidate these associations. The Family Stress Model (FSM) also provides important theoretical framing since it distinguishes between objective economic conditions (e.g., income, employment, earnings) and families’ subjective experiences of economic pressure (Conger et al., 1992) which are both associated parental psychological distress. In this study, we draw on the FSM to examine both structural economic indicators and subjective economic pressure as predictors of depressive symptoms.

Edwards et al. (2021) conducted a systematic review and meta-analysis of ten studies examining 11 risk factors for postpartum depressive symptoms among Latinas, finding a large effect size for partner/father social support, medium effect sizes for prenatal depression and recent intimate partner violence (IPV), and small effect sizes for education, economic stress, general social support, and past IPV. In their review, Edwards et al. (2021) found no significant effects for age, marital status, number of children, or acculturation. However, there were not enough studies to evaluate the risk factors of acculturative stress, immigration status, and discrimination (Edwards et al., 2021). As the research on this topic is nascent, Edwards et al. (2021) noted that risk factors were often measured very differently across studies.

There is a lack of research on depression among mothers in Head Start, especially among Latina mothers. Only one prior study has focused on maternal depressive symptoms among Latina Head Start mothers (Lee & Rispoli, 2017), yet this study did not examine socioeconomic, psychosocial, health, and cultural factors in understanding risk for maternal depression.

## The Current Study

The current study aimed to apply the FSM and an adaptation of the model articulated by Lara-Cinisomo et al. (2016) to Latina Head Start mothers by examining whether a range of contextual and cultural stressors were associated with depressive symptoms. In this study, Lara-Cinisomo’s conceptualization of contextual stressors is further refined by examining the impact of both socioeconomic and psychosocial/health factors and including a wide range of factors in each domain, with a particular focus on objective and subjective indicators of economic stress. This study also adds to the existing literature by examining associations with maternal trauma exposure, including both current IPV and lifetime trauma exposure. We hypothesized that a range of socioeconomic and psychosocial/health factors would significantly predict maternal depressive symptoms based on prior literature. We also had two exploratory hypotheses. The first was that insurance status might be an important additional aspect of economic hardship for Latina mothers, particularly if they are undocumented. We also hypothesized that cultural factors like language and country of origin could predict depressive symptoms among Latina mothers, though prior lite1$0oxy_rature is mixed.

## Materials & Methods

### Participants

Data for this study were drawn from baseline assessments collected as part of a larger cluster-randomized intervention study that provided a mental health treatment for low-income mothers experiencing depression (withheld for blind review). Participating Head Start sites (*n* = 25) were randomized to study conditions prior to participant recruitment. The present analyses pool participants across all study conditions and reflect pre-intervention baseline characteristics only. A total of 2,902 mothers were approached for initial depression screening and 2,466 consented. Following site-level randomization, mothers who screened positive for depressive symptoms (*n* = 457) were prioritized for enrollment into the study. Mothers who did not screen positive (*n* = 2,009) were enrolled gradually to meet target sample sizes for comparison purposes. In total, 208 mothers provided informed consent for study enrollment across intervention, control, and comparison groups. Of these, 163 mothers identified as Latina and comprise the analytic sample for the present study.

Eligibility criteria for the present analyses included: (1) mother of a Head Start child, (2) consented to the parent study, (3) spoke English or Spanish, and (4) self-reported Latina ethnicity. Mothers included in this analysis answered “Hispanic/Latino” to the question, “Which category best describes you? (check all that apply).” Mothers were also asked “Which of the below best describes you?” Response options included Mexican, Salvadorian, Guatemalan, Nicaraguan, Honduran, Puerto Rican, Cuban, etc. Characteristics of the participants (*n* = 163) are presented in Table 1. On average, mothers in the sample were 32.7 years of age (*SD* = 6.3). Overall, 41.4% of mothers were born in Mexico and 25.2% were born in the United States. Among US-born mothers (*n* = 41), 76% identified as Mexican American and 15% identified as Salvadorian American. Almost half of mothers in the sample reported not completing high school and less than 10% reported completing college or advanced degree. Over 60% of families reported having annual household incomes below $35,000.

**Table 1:** Characteristics of participants (n=163)

*Maternal demographic characteristics*	
Age, mean (SD), years	32.7 (6.3)
Country of Birth, n (%)	
Mexico	67 (41.1)
United States	41 (25.2)
El Salvador	23 (14.1)
Guatemala	18 (11.0)
Honduras	10 (6.1)
Other Central/South American country	4 (2.5)
Ethnicity, n (%)^1^	
Mexican/Mexican American	97 (59.5)
Salvadorian	31 (19.0)
Guatemalan	19 (11.7)
Honduran	8 (4.9)
Puerto Rican	2 (1.2)
Ecuadorian	1 (0.6)
Cuban	1 (0.6)
Other Central American	1 (0.6)
Other South American	1 (0.6)
Spain/other European country	1 (0.6)
Refused	2 (1.2)
Preferred language, n (%)	
Spanish	95 (58.3)
English	68 (41.7)
Educational level, n (%)	
Less than high school	77 (47.2)
High school diploma	48 (29.4)
Some college / trade school	25 (15.3)
College degree or higher	13 (8.0)
Household income, n (%)	
Under $15,000	29 (17.8)
$15,000 - $24,999	52 (31.9)
$25,000 - $34,999	22 (13.5)
$35,000 - $49,999	12 (7.4)
$50,000 and above	3 (1.8)
Refused	45 (27.6)
*Child demographic characteristics*	
Age, mean (SD), years	4.2 (0.6)
Sex, n (%)	
Male	85 (52.1)
Female	78 (47.9)

^1^Respondents could endorse more than one ethnicity.

### Procedures

Mothers were recruited into the parent intervention study (withheld for blind review) and completed baseline measures between 2015 and 2018 in Los Angeles, California. As part of the intake process, all mothers enrolling in Head Start sites operated by a large social service agency were screened for depression using the CES-D. Mothers were typically screened in their homes as part of the standard intake procedure. Members of the research team obtained written consent from participants, and the study was approved by the university’s Institutional Review Board and therefore performed in accordance with the ethical standards laid down in the 1964 Declaration of Helsinki and its later amendments. After obtaining consent, members of the research team administered a series of baseline questionnaires to mothers in a setting of their choosing.

### Measures

#### Maternal Depressive Symptoms

Maternal self-report of current depressive symptoms was assessed using the 20-item Center for Epidemiology Studies Depression Scale (CES-D; Radloff, 1977). Studies show that the CES-D has high reliability and validity (Cosco et al., 2017; Radloff, 1977). The Spanish version of the CES-D used in this study was psychometrically validated by Ruiz-Grosso et al. (2012). A sum score on the CES-D was used to analyze significant predictors of depressive symptoms. A cut-off score of 16 was used to calculate the percentage of the sample that had clinically significant levels of depressive symptomatology (Lewinsohn et al., 1997).

#### Socioeconomic Indicators

About 28% of families were missing data on household income, so maternal report of receiving income from earnings was included. Maternal report of family economic pressure was assessed using eight items based on prior measures of economic pressure and financial strain (Conger et al., 1992). Mothers answered yes or no to items assessing the extent to which the family experienced difficulty paying for a variety of expenses in the past six months. Items were summed to create a scale ranging from 0 to 8. Maternal education level was captured on a continuous scale from 0 = never attended school to 20 = 4 years or more after bachelor’s degree. Maternal insurance status was measured by self-report of public, private, or no insurance. Number of children was indicated by maternal self-report of the total number of children in the home. Maternal partner status and employment status were assessed using dichotomous (yes/no) measures.

#### Psychosocial and Health Factors

Maternal report of lifetime exposure to trauma was assessed using the Trauma History Questionnaire (THQ), a well-validated and reliable scale that assesses adverse and traumatic experiences across several domains (Hooper et al., 2011). The THQ has been translated into Spanish and used in prior studies with low-income women (Heilemann et al., 2005). In this paper, the total number of traumatic events was used, since research shows that additive trauma experiences are associated with worse psychological, emotional, and interpersonal problems (Van Der Kolk et al., 1996).

Maternal IPV exposure was assessed using the revised Conflict Tactics Scales (CTS2) (Straus et al., 1995), which includes subscales for verbal and physical IPV. The CTS2 has been used widely across different socioeconomic and racial/ethnic groups and has well-established psychometric properties (Straus, 1995). The CTS2 has been translated into Spanish and demonstrates good internal consistency (Connelly et al., 2005). For this analysis, subscales were re-coded to capture the severity of the type of abuse, with 0 = mother did not report verbal or physical IPV, 1 = mother reported only verbal IPV, or 2 = mother reported both verbal and physical IPV. All mothers in this sample who reported exposure to physical IPV also reported exposure to verbal IPV.

Maternal report of social support was assessed using an adapted version of the Duke-UNC Functional Social Support Questionnaire (FSSQ) (Broadhead et al., 1988). This measure has demonstrated good reliability and construct validity (Broadhead et al., 1988). Items were translated into Spanish by the research team. A sum score on the FSSQ was used.

Maternal age was reported in years. For physical health problems, mothers indicated whether they had been diagnosed with high blood pressure, diabetes, asthma or other breathing problems, or another health problem (scores ranged from 0 to 5). Maternal prior history of depression was assessed using a dichotomous (yes/no) measure. 

#### Cultural Variables

English versus Spanish language preference was captured based on the language mothers chose to complete the maternal depression screener. Maternal nativity was also captured (U.S.-born versus foreign-born).

### Statistical Analysis

Data analysis was conducted in SPSS and SAS. Hierarchical regression models were used to examine whether socioeconomic factors (block 1), psychosocial and health factors (block 2) and cultural factors (block 3) explained a statistically significant amount of variance in maternal depressive symptoms when entering the model in that order. The analysis was conducted using the complete cases (*n* = 163) so the three models can be considered as nested models. Six participants with missing data on the Trauma History Questionnaire, four participants with missing data on the Conflict Tactics Scale, and one participant with missing education were excluded from the original sample (*n* = 174) in this step. Bivariate correlations were examined to assess associations among variables and evaluate potential multicollinearity prior to hierarchical regression analyses. Assumptions of linear regression, including linearity, homoscedasticity, normality of residuals, and absence of multicollinearity, were evaluated and met.

## Results

Overall, 38% of Latina Head Start mothers in this sample met the clinical cut-off score of 16 on the CES-D indicating clinically significant levels of depressive symptomatology. Mothers in the sample endorsed high rates of cumulative lifetime trauma exposure, with 63% experiencing general disasters, 48% experiencing crime-related events, 23% experiencing sexual abuse and violence, 20% experiencing the death of a loved one, and 18% experiencing general physical abuse. Bivariate associations and descriptive statistics are included in tables S1 and S2. In the final multivariate model, receiving income from earnings was negatively associated with depressive symptoms (*β*=−0.14, *p*=.029). Lifetime trauma exposure and current exposure to verbal/physical IPV were both positively associated with maternal depressive symptoms (*β* = 0.22, *p*=.001 and *β* = 0.22, *p*=.008, respectively). Endorsing a prior history of depression was also positively associated with maternal depressive symptoms (*β* = 0.16, *p*=.009). However, report of social support was negatively associated with maternal depressive symptoms (*β*=−0.38, *p*<.001). The second model showed significant improvement from the first model (∆*R*^*2*^=0.37, *p*<.001), and there was no significant improvement in the third model after the cultural factors were added. The final model explained 56% of the variance in maternal depressive symptoms (Table 2).

**Table 2:** Results of hierarchical regression models predicting Latina mothers’ depressive symptoms (*n* = 163)

	Model 1	Model 2	Model 3
*Block 1: Socioeconomic factors*			
Maternal education level (yrs)	0.01	− 0.04	− 0.03
Maternal employment status (yes/no)	0.02	− 0.01	0.01
Maternal insurance status			
Public	0.07	− 0.01	0.01
Private	0.02	0.06	0.06
None	–	–	–
Family receives income from earnings (yes/no)	− 0.16*	− 0.14*	− 0.14*
Family economic pressure	0.41*	0.08	0.08
Number of children in household	− 0.04	0.06	0.06
*Block 2: Psychosocial and health factors*			
Maternal age (yrs)		− 0.12*	− 0.13
Living with a partner (yes/no)		− 0.11	− 0.11
Total number of physical health problems		0.10	0.10
Maternal lifetime trauma exposure (THQ)		0.22*	0.22*
Maternal intimate partner violence (CTS) exposure			
Verbal abuse without physical abuse		0.11	0.11
Physical and verbal abuse		0.23*	0.22*
No verbal or physical abuse		–	–
Social support (Duke-UNC FSSQ)		− 0.38*	− 0.38*
Prior history of depression (yes/no)		0.16*	0.16*
*Block 3: Cultural factors*			
Foreign-born (yes/no)			0.01
Language preference (English/Spanish)			0.01
*R* ^*2*^	0.18	0.56	0.56
*R*^*2*^ *increase*		0.37	0.00
*F-statistic*		13.73	0.01
*P-value*		< 0.001	0.99

*p<.05

## Discussion

This study examined whether a comprehensive set of socioeconomic, psychosocial, health, and cultural factors were associated with maternal depressive symptoms among Latina Head Start mothers. Findings showed that several psychosocial factors were strongly associated with depressive symptoms, socioeconomic factors were moderately associated, and cultural factors showed no associations.

Overall, 38% of Latina mothers in this sample reported clinically significant levels of depressive symptoms, which is similar to prior studies among the general population of Head Start mothers that reported rates of 36% (Baker, 2018) and 41% (Lanzi et al., 1999). This finding indicates a high rate of clinically elevated depressive symptoms in this sample, particularly since a nationally representative study found that only 14.5% of mothers of U.S. kindergartners met clinical cut-off on the CES-D (Vericker, 2015). However, this high rate is likely due to the design of the parent study, and future research should explore the rate of maternal depression among Head Start mothers by race and ethnicity using representative sampling methods.

In terms of socioeconomic factors, receiving income from earnings predicted lower maternal depressive symptoms. This finding aligns with the Family Stress Model and Lara-Cinisomo model of postpartum depression, which emphasize how structural and socioeconomic vulnerabilities increase mental health concerns. Additionally, this finding demonstrates the importance of distinguishing between the type of economic support the family is receiving and calls for additional investigation regarding this association. For example, Lanzi et al. (1999) examined correlates of maternal depressive symptoms among U.S. Head Start mothers and found that receiving any of three public benefits significantly increased the risk of maternal depressive symptoms. However, family economic pressure became insignificant after adding psychosocial and health correlates to the model, showing that subjective report of economic pressure was explained by these other stressors. Results showed no significant associations for insurance status, which was surprising since prior research has shown that lack of insurance is associated with increased odds of mood and anxiety disorders (Sareen et al., 2016).

In terms of psychosocial and health factors, results showed that higher lifetime maternal trauma exposure, exposure to both current verbal and physical IPV, and a prior history of depression significantly predicted higher maternal depressive symptoms, whereas higher levels of social support predicted lower depressive symptoms. In contrast, living with a partner, total number of health problems, and current exposure to verbal IPV alone were not significant predictors of maternal depressive symptoms. This study included a more complete range of important psychosocial and health factors in one analysis than prior studies and demonstrates key new findings. For example, in their meta-analysis of predictors of maternal depression among Latina mothers, Edwards et al. (2021) examined findings related to current and past IPV but did not include general lifetime trauma exposure. In the only prior study of depression among Latina mothers that included both types of trauma exposure, Valentine et al. (2011) found that current IPV, but not past non-IPV trauma, was significant in a multivariate model. In the current study, lifetime trauma exposure and current verbal/physical IPV uniquely contributed to maternal depressive symptoms, and Latina Head Start mothers reported high levels of exposure to general disaster events, crime, and sexual abuse or violence over the course of their lifetimes. A prior history of depression was also a significant predictor of higher maternal depressive symptoms. This echoes previous review studies focusing on both the general population (Al-abri et al., 2023) and Latina mothers (Edwards et al., 2021).

As hypothesized, low social support was a significant predictor of maternal depressive symptoms, which aligns with prior work among both the general U.S. population and Latina mothers (Al-abri et al., 2023; Edwards et al., 2021; Howell et al., 2005). The effect size in the current study was larger than in the prior systematic review examining correlates of depression among Latina mothers (Edwards et al., 2021) and was the strongest predictor of maternal depressive symptoms in this sample. However, we were unable to differentiate between social support provided by partners and social support provided by family, friends, and others. Edwards et al. (2021) distinguished between these two types and found a stronger effect for partner support.

In terms of cultural factors, similar to Edwards et al. (2021) and Ponting et al. (2020), we found no relationship between our two variables of acculturation and maternal depressive symptoms. However, both of our measures were binary indicators that were used to proxy acculturation. While there are arguments against using these types of measures due to their focus on single aspects of the acculturation process (Lopez-Class et al., 2011), other research has validated the use of single-item language variables as a measure of acculturation (Mills et al., 2014). Overall, findings from this study show that for this sample, psychosocial factors were more predictive of maternal mental health than these two measures of acculturation.

This study has several strengths and is an important contribution to the literature. First, it focuses on low-income Latina mothers of preschool children, who may still experience high rates of depressive symptoms. Second, it combines several important correlates of maternal depression identified in prior research on the U.S. general population and Latina mothers in a single analysis, including a range of socioeconomic, psychosocial, and health factors, and includes both subjective and objective measures of economic stress. Third, it examines the effects of cultural factors for Latina mothers, which have demonstrated mixed findings in prior literature. Fourth, it examined the potential role of insurance status, which has not been included in prior studies. Lastly, it utilized high quality measures of two types of trauma exposure (lifetime trauma and current IPV) to build on prior research.

This study has several limitations. First, analyses relied on cross-sectional baseline data and therefore cannot address causality. Second, the proportion of Latina mothers with clinically significant depressive symptoms is likely overestimated due to selection bias, as recruitment in the parent study prioritized mothers who screened positive for depression. Third, depressive symptoms and related constructs were assessed via self-report and may be subject to social desirability bias, particularly given the sensitive nature of mental health reporting in a service-based context. Finally, the study was limited by measurement constraints, including reliance on proxy indicators of acculturation, lack of partner-specific social support measures, and a sample composed predominantly of Mexican American mothers, which precluded subgroup analyses.

This study has several practice and policy implications. Findings highlight several potential contributors to elevated depressive symptoms among Latina Head Start mothers. Receiving income from earnings, a socioeconomic factor, was negatively associated with depressive symptoms, whereas psychosocial stressors like lifetime trauma exposure, current IPV exposure, a prior history of depression, and lack of social support were positively associated with depressive symptoms. Although data were collected prior to the COVID-19 pandemic and current immigration context, the structural and psychosocial inequities examined remain highly relevant and likely represent a conservative estimate of current stressors experienced by low-income Latina mothers. Therefore, interventions aimed at treating depressive symptoms among this population should follow trauma-informed principles throughout the helping process and include specific content related to processing trauma. Additionally, interventions should focus on identifying and promoting sources of social support for Latina mothers. Second, although several socioeconomic correlates were not significant in this study, income from earnings was associated with lower depressive symptoms. This indicates that socioeconomic status may still be an important contributor, and interventions aimed at reducing depressive symptoms among this population should not only focus on sequelae related to depression, but potential root causes related to income.

## Conclusion

This is the first study to examine a wide range of potential correlates of maternal depressive symptoms among Latina Head Start mothers. Findings showed a high rate of clinically elevated maternal depressive symptoms among this sample. Receiving income from earnings and reporting higher levels of social support were negatively associated with maternal depressive symptoms, whereas higher levels of lifetime trauma exposure, current exposure to physical and verbal IPV, and endorsing a prior history of depression were positively associated with depressive symptoms. There were no significant associations for cultural factors. Although research and intervention tend to focus on the perinatal period, this study highlights the importance of considering the impact of depressive symptoms among Latina mothers of preschool age children. Findings also indicate the need for cross-system interventions that address both structural and psychosocial contributors to depression when intervening with Latina mothers.

## Supplementary Information

Below is the link to the electronic supplementary material.

Supplementary material 1 (DOCX 20.0 kb)

## Data Availability

The participants of this study did not give written consent for their data to be shared publicly, so supporting data is not available.

## Footnotes

1 The postpartum period typically refers to the first three or six months after the birth of a child. Postpartum depression refers to depression that occurs during that period. The term perinatal depression is also commonly used and includes depression during pregnancy and the year following childbirth (O’Hara, 2014).

## Code Availability

Not available.
