# Supplementary material for: Correlates of Maternal Depressive Symptomatology Among Latina Head Start Mothers
Source: Matern Child Health J. 2026 Apr 10;30(4):490–7. doi: 10.1007/s10995-026-04253-4 (PMC13135565; doi:10.1007/s10995-026-04253-4)
Supplement: Supplementary file 1 — Supplementary material 1 (DOCX 20.0 kb) [file 10995_2026_4253_MOESM1_ESM.docx]

| Table S1. Correlations between study variables (*n*=163). | | | | |  |  |  |  |  |  |  |  |  |  |  |  |  |  |
| --- | --- | --- | --- | --- | --- | --- | --- | --- | --- | --- | --- | --- | --- | --- | --- | --- | --- | --- |
| Variable | 1 | 2 | 3 | 4 | | 5 | 6 | 7 | 8 | 9 | 10 | 11 | 12 | 13 | 14 | 15 | 16 | 17 |
| 1. Level of education | - |  |  |  | |  |  |  |  |  |  |  |  |  |  |  |  |  |
| 2. Employment status | .26** | - |  |  | |  |  |  |  |  |  |  |  |  |  |  |  |  |
| 3. Insurance type (public) | .03 | -.22** | - |  | |  |  |  |  |  |  |  |  |  |  |  |  |  |
| 4. Insurance type (private) | .21** | .14 | -.38** | - | |  |  |  |  |  |  |  |  |  |  |  |  |  |
| 5. Income from earnings | .19* | .19* | .09 | .06 | | - |  |  |  |  |  |  |  |  |  |  |  |  |
| 6. Economic pressure | -.08 | .04 | -.12 | -.15 | | -.04 | - |  |  |  |  |  |  |  |  |  |  |  |
| 7. Number of children | -.23** | -.14 | -.01 | -.10 | | -.11 | .17* | - |  |  |  |  |  |  |  |  |  |  |
| 8. Maternal age | -.17* | -.01 | -.02 | -.01 | | .02 | .03 | .26** | - |  |  |  |  |  |  |  |  |  |
| 9. Living with partner | -.07 | -.05 | -.05 | .08 | | .13 | -.09 | .15 | .04 | - |  |  |  |  |  |  |  |  |
| 10. Total health problems | .05 | .06 | .13 | -.08 | | .11 | .01 | -.02 | .08 | .03 | - |  |  |  |  |  |  |  |
| 11. Lifetime trauma | .24** | .13 | .10 | -.13 | | .23** | .29** | -.08 | .09 | -.10 | .19* | - |  |  |  |  |  |  |
| 12. IPV-Physical | .04 | .13 | -.02 | .03 | | -.09 | .16* | -.06 | -.10 | -.36** | .12 | .15* | - |  |  |  |  |  |
| 13. IPV-Verbal | .05 | .11 | .06 | -.04 | | -.08 | .35** | -.15 | -.09 | -.47** | .14 | .27** | .69** | - |  |  |  |  |
| 14. Social support | .16* | .07 | .06 | .10 | | .14 | -.36** | -.09 | -.01 | .11 | -.17* | -.17* | -.18* | -.31** | - |  |  |  |
| 15. HHistory of depression | .05 | .08 | .11 | -.10 | | .06 | .27** | -.06 | -.01 | -.07 | .23** | .24** | .10 | .23** | -.26** | - |  |  |
| 16. Foreign-born | -.29** | -.04 | -.21** | -.23** | | -.14 | .16* | .10 | .35** | .08 | -.07 | -.02 | .02 | -.02 | -.08 | -.11 | - |  |
| 17. Spanish preference | .23** | .05 | .04 | .10 | | .13 | -.11 | -.08 | -.37** | -.16* | -.04 | -.02 | .06 | .03 | .08 | .05 | -.57** | - |
| 18. Depressive symptoms | -.03 | .01 | .00 | -.07 | | -.16* | .40** | .03 | -.09 | -.26** | .22** | .35** | .28* | .55** | -.59** | .38** | -.02 | .01 |
| *Note:* **p*<.05, ***p*<.01 |  |  |  |  | |  |  |  |  |  |  |  |  |  |  |  |  |  |

| Table S2: Descriptive statistics for study variables. | | |
| --- | --- | --- |
| Variable | *M* | *SD* |
| 1. Level of education | 10.67 | 3.27 |
| 2. Employment status | - | - |
| 3. Insurance type (public) | - | - |
| 4. Insurance type (private) | - | - |
| 5. Income from earnings | - | - |
| 6. Economic pressure | 1.67 | 1.81 |
| 7. Number of children | 2.63 | 1.26 |
| 8. Maternal age | 32.67 | 6.25 |
| 9. Living with partner | - | - |
| 10. Total health problems | 0.29 | 0.59 |
| 11. Lifetime trauma |  |  |
| 12. IPV-Physical | 1.55 | 5.26 |
| 13. IPV-Verbal | 5.36 | 6.34 |
| 14. Social support | 36.33 | 10.01 |
| 15. HHistory of depression | - | - |
| 16. Foreign-born | - | - |
| 17. Spanish preference | - | - |
| 18. Depressive symptoms | 13.42 | 11.75 |
